# SEOM clinical guidelines for the treatment of advanced prostate cancer (2020)

**DOI:** 10.1007/s12094-021-02561-5

**Published:** 2021-02-24

**Authors:** A. González del Alba, M. J. Méndez-Vidal, S. Vazquez, E. Castro, M. A. Climent, E. Gallardo, E. Gonzalez-Billalabeitia, D. Lorente, J. P. Maroto, J. A. Arranz

**Affiliations:** 1 Medical Oncology Department, Hospital Universitario Puerta de Hierro-Majadahonda, Joaquin Rodrigo 2, Majadahonda, 28222 Madrid, Spain; 2 Medical Oncology Department, Hospital Universitario Reina Sofía, Maimonides Institute for Biomedical Research of Córdoba (IMIBIC), Córdoba, Spain; 3 Medical Oncology Department, Hospital Universitario Lucus Augusti, Lugo, Spain; 4 Medical Oncology Department, Hospital Universitario Virgen de la Victoria y Regional de Mälaga, Málaga, Spain; 5 Medical Oncology Department, Fundación Instituto Valenciano de Oncología, València, Spain; 6 Medical Oncology Department, Parc Taulí Hospital Universitari, Institut d’Investigació i Innovació Parc Taulí I3PT, Universitat Autònoma de Barcelona, Sabadell, Spain; 7 Medical Oncology Department, Hospital Universitario Doce de Octubre, Instituto Imas12, Madrid, Spain; 8 Universidad Católica San Antonio de Murcia (UCAM), Murcia, Spain; 9 Medical Oncology Department, Hospital Provincial de Castellón, Castellon, Spain; 10 Medical Oncology Department, Hospital Universitari Santa Creu i San Pau, Barcelona, Spain; 11 Medical Oncology Department, Hospital General Universitario Gregorio Marañón, Madrid, Spain

**Keywords:** Androgen, Castration, Molecular, Biomarkers, Research

## Abstract

The treatment of advanced prostate cancer has evolved due to recent advances in molecular research and new drug development. Dynamic aberrations in the androgen receptor, DNA repair genes, PTEN-PI3K, and other pathways drive the behavior of advanced prostate cancer allowing a better selection of therapies in each patient. Tumor testing for BRCA1 and BRCA2 is recommended for patients with metastatic prostate cancer, also considering a broad panel to guide decisions and genetic counseling. In symptomatic metastatic patients, castration should be stared to palliate symptoms and prolong survival. In high-risk or high-volume metastatic hormone-naïve patients, castration should be combined with docetaxel, abiraterone, enzalutamide or apalutamide. Radiotherapy to the primary tumor combined with systemic therapy is recommended in low-volume mHNPC patients. In patients with non-metastatic castration-resistant tumors, risk stratification can define the frequency of imaging. Adding enzalutamide, darolutamide or apalutamide to these patients prolongs metastasis-free and overall survival, but potential adverse events need to be taken into consideration. The choice of docetaxel, abiraterone or enzalutamide for treating metastatic castration-resistant patients depends on previous therapies, with cabazitaxel being also recommended after docetaxel. Olaparib is recommended in BRCA1/BRCA2 mutated castration-resistant patients after progression on at least one new hormonal therapy. Aggressive variants of prostate cancer respond to platinum-based chemotherapy. To optimize treatment efficiency, oncologists should incorporate all of these advances into an overall therapeutic strategy.

## Introduction

Prostate cancer is a major health issue in Western countries, representing the most frequent cancer and the fifth cause of cancer-related deaths among males. According to GLOBOCAN, new 1.3 million prostate cancer cases were diagnosed in the world and accounted for 359,000 deaths in 2018 (3.8% of cancer mortality) [1]. In Spain, the incidence in 2020 was 35,126 new cases with an estimated mortality in 2018 of 5841 cases [2].

Most cases present at an early stage and often have an indolent course. However, less than 10% of cases will have metastatic disease onset and it is estimated that up to one-third of patients will develop eventual metastatic disease at some point of their disease course.

## Methodology

This guideline is focused on the systemic treatment of advanced prostate cancer and has been developed based on the consensus of 10 genitourinary medical oncologists, designed by the Spanish Society of Medical Oncology (SEOM) and the Spanish Oncology Genitourinary Group (SOGUG), with the purpose of reviewing and summarizing the available evidence regarding the management of MPC, as well as generating evidence-based statements on diagnostics and therapeutic strategies. To be in accordance with previous SEOM guidelines [3], the rating system for quality of the evidence (I–III) and strength of the recommendation (A–E) criteria summarized in Table 1 has been followed [4]. Systematic reviews and meta-analysis of well-designed randomized clinical trials, although not included in the table, have also been considered as level of evidence I. Recommendations are based on current evidence, but the local regulatory status of drugs and procedures should be considered by the reader.

**Table 1:** Levels of evidence and grades of recommendation

Category, grade	Criteria
Quality of evidence	
I	Evidence from at least 1 properly randomized, controlled trial *
II	Evidence from at least 1 well-designed clinical trial without randomization, from cohort or case-controlled analytical studies (Preferably from more than 1 centre), or from multiple time-series or dramatic results from uncontrolled experiments
III	Evidence from opinions of respected authorities based on clinical Experience, descriptive studies, or reports of expert committees
Strength of recommendation	
A	Both strong evidence of efficacy and substantial clinical benefit Support recommendation for use. Should always be offered
B	Moderate evidence of efficacy—or strong evidence of efficacy but only limited clinical benefit—supports recommendation for use Should generally be offered
C	Evidence of efficacy is insufficient to support a recommendation For or against use, or evidence for efficacy might not outweigh adverse consequences (e.g., drug toxicity, drug interactions) or Cost of the chemoprophylaxis or alternative approaches Optional
D	Moderate evidence of lack of efficacy or of adverse outcome supports a recommendation against use Should generally not be offered
E	Good evidence of lack of efficacy or of adverse outcome supports a recommendation against use Should never be offered

## Molecular features and biomarkers.

In recent years, several studies have depicted the molecular landscape of advanced prostate cancer revealing that most patients with advanced prostate cancer harbor actionable alterations in some specific pathways [5].

AR is upregulated in up to 85% of patients, showing dynamic aberrations upon treatment pressure, such as AR amplification, AR mutations, amplification of a non-coding enhancer of AR, or alternative splicing variants of AR (AR-V), the most common being AR-V7, that associate resistance to anti-androgen treatments. Some of these alterations can be detected both in tumor and in circulating tumor cells (CTCs) or tumor DNA and are associated with worse evolution on anti-androgen treatments [6], whereas taxanes seem to retain activity [7–9]. Due to the dynamic nature of AR aberrations and the changing therapeutic landscape, these biomarkers need to be clinically qualified in each clinical indication [10].

There is a crosstalk between the AR and the PTEN-PI3K pathways. Loss of PTEN, a tumor suppressor gene, is present in 40–50% of MPC, and it is associated with anti-androgen resistance and poor survival [11, 12]. Several AKT-inhibitors are currently being explored in clinical trials using this biomarker.

Approximately 23% of metastatic prostate cancers harbor mutations in DNA-Damage Repair (DDR) genes [4], including *BRCA2, BRCA1, ATM, CDK12*, or *PALB2* among others. Importantly, half of the DDR mutations identified in prostate tumors are also present in the germline [13] and represent an inherited cancer predisposition. Germline mutations in *BRCA2* and *BRCA1* are associated with increased incidence and aggressiveness of prostate cancer [14], and have been identified as biomarkers for platinum therapy [15] and PARP inhibitors [16].

Genomic events leading to a mismatch repair (MMR) deficiency and microsatellite instability (MSI) have been reported in 3.1% of prostate cancer cases, mostly affecting MSH2 [17], and associated with increased risk of prostate cancer and higher mutational tumor burden [18]. Finally, loss of TP53 is observed in up to 50% of lethal prostate cancers and when associated with loss of RB, is associated with divergent neuroendocrine differentiation, a more aggressive AR-independent disease [19].

Circulating tumor cells (CTCs) can be identified in the circulation of metastatic patients. CTCs detection has demonstrated to be prognostic [20] and a good intermediate endpoint in clinical trials fulfilling the prentice criteria [21]. In addition, CTC molecular characterization brings the possibility to analyze circulating tumor clones. Its use outside clinical research is limited due to the high cost and low implication in treatment decisions.

Given the above landscape of actionable specific pathways, the assessment of predictive biomarkers of response should be steadily incorporated in clinical practice, particularly in patients with castration-resistant prostate cancer (CRPC). However, several questions remain open such as how to optimize tissue quality for genetic testing, the use of samples from the primary tumor to take decisions at the time of castration resistance, the validity of liquid biopsy, and the genes that should be included in the panels for tumor profiling as further evidence emerges.

### Recommendations

- Tumor testing for *BRCA1* and *BRCA2* is recommended for patients with metastatic prostate cancer [I, A].
- The assessment of other potential biomarkers of response should be steadily incorporated in clinical practice as further evidence emerges. Consider a broad panel including other HHR and MMR genes (and/or perform MSI assessment) (II, B).

## Therapeutic approach in metastatic hormone-naive prostate cancer patients (mHNPC)

- Androgen deprivation therapy (ADT): strategies, indications, and duration
  Primary androgen deprivation therapy (ADT) is mandatory in the first-line treatment for mHNPC. Nearly 90% of patients respond to ADT with a median progression-free survival (PFS) of 12–24 months. There is no clear evidence in favor of any type of ADT (orchiectomy vs LHRH analog vs LHRH antagonist). The only exception are patients with impending or high-risk spinal cord compression for whom either a bilateral orchidectomy, or LHRH antagonists are the preferred options to avoid testosterone flare that could worsen symptomatology of the patients. The combination of anti-androgen blockade with a first-generation non-steroidal anti-androgen is recommended during the first month to avoid androgen flare. Longer duration appears to offer a small 5-year overall survival (OS) advantage (less than 5%) vs surgical castration or LHRH agonists, that should be balanced against the higher toxicity associated with long-term anti-androgen use [22]. As indicated below, the addition of newer hormonal therapies or docetaxel has improved these results.
  ADT is the cornerstone treatment for all metastatic patients. Once the diagnosis is established, immediate initiation is mandatory in symptomatic patients and should be recommended to asymptomatic ones, after discussion with the patient on the risk and benefits of the treatment. Although controversy still exists concerning initial vs deferral initiation of ADT, current evidence favors early over delayed ADT in terms of survival and other oncological outcomes [23].
  Several studies have elucidated the role of intermittent androgen deprivation (IAD) in mHNPC patients [24]. In the SWOG 9346 trial, non-inferior OS with IAD could not be completely ruled out. Although in different trials, there is a trend to a better quality of life with IAD and a protective effect against bone loss, metabolic syndrome and cardiovascular problems, the lack of any survival benefit suggest that this treatment should only be considered as an option in a well-informed patient bothered by significant side-effects. Usually, patients are treated with an induction period of ADT for 9 months. If no clinical progression and PSA response < 4 ng/ml are achieved, therapy is stopped. Patient should be strictly followed up every 3–6 months. If PSA raises over 10–20 ng/ml, treatment with AT is resumed at least for 3–6 months. Treatment is stopped again if the patient shows no clinical progression and PSA response. Subsequent cycles of treatment are based on the same criteria until the first sign of castration resistance becomes apparent [25].
  Recommendations:
  In symptomatic metastatic patients, ADT should be offered immediately to palliate symptoms and prolong survival (I, A).
  Deferred ADT could be considered in selected well-informed asymptomatic patients to minimize long-term adverse effects (II,A).
  Combination of LHRH with first generation anti-androgens for longer than one month to avoid androgen flare, does not offer clinical benefit (I, D).
- Combination of ADT and chemotherapy or new hormonal agents
  Recently, the combination of *ADT with* *docetaxel, abiraterone, apalutamide, or enzalutamida*, or enzalutamida has demonstrated to improve OS in mHNPC in selected patients and it has been incorporated into most clinical practice guidelines. Optimal patient selection for this approach is not well-established.

### *b1. ADT plus Docetaxel in mHNPC*

The addition of docetaxel for mHNPC was studied in three phase III trials. The CHAARTED study randomized 790 patients to receive ADT alone or in combination with docetaxel 75 mg/m^2^ every 21 days for 6 cycles. Docetaxel improved OS (median 58 versus 47 months, hazard ratio (HR) 0.72; 95% CI 0.59–0.89). Benefit was more significant (median 51 versus 34 months, HR 0.63, 95% CI 0.50–0.79) for men with high-volume metastatic disease (defined as the presence of visceral metastases or at least four bone lesions with at least one outside the vertebral bodies and pelvis) [26, 27]. STAMPEDE was a multi-arm, multi-stage phase III study designed to test whether adding additional treatments to ADT improved OS. It included patients with M0 and M1 disease. Patients were randomized to ADT alone (*n* = 1184) or in combination with docetaxel 75 mg/m^2^ every 21 days with prednisone 10 mg daily for six cycles (*n* = 592). The addition of docetaxel in M1 patients significantly improved OS compared to ADT alone (HR 0.76; 95% CI 0.62–0.92) [28]. The benefit of docetaxel for OS was similar when combined with zoledronic acid (HR 0.79; 95% CI 0.66–0.96). The third study, GETUG-AFU15, randomized 385 patients with mHNPC to receive ADT or ADT plus docetaxel 75 mg/m^2^ every 21 days for 9 cycles. Patients in the docetaxel arm had improved PSA, PFS and radiographic PFS (rPFS), but without an OS benefit (HR 1.01; 95% CI 0.75–1.36) [29]. The meta-analysis of CHAARTED, STAMPEDE, and GETUG-AFU 15 confirmed the benefit of docetaxel and ADT in OS regardless of the disease volume (HR 0.77; 95% CI 0.68–0.87) [30].

### *b2. ADT plus Abiraterone—prednisone in mHNPC*

Two randomized clinical trials (LATITUDE and STAMPEDE) have shown that the combination of abiraterone plus ADT significantly prolongs OS and secondary endpoints in patients with mHNPC.

In the LATITUDE trial, 1199 men with newly diagnosed mHNPC were randomly assigned to ADT plus abiraterone and prednisone or placebo. All patients had “high-risk” disease (at least two of these high-risk factors: Gleason score ≥ 8, three or more bone lesions, or the measurable visceral metastasis). After a median follow-up of 52 months, OS was significantly increased in the arm of abiraterone plus prednisone (median OS 53.3 versus 36.5 months, HR 0.66, 95% CI 0.56–0.78) [31, 32].

In the STAMPEDE trial, 1917 men were randomly assigned to ADT plus abiraterone and prednisolone or to ADT alone. The patient population was heterogeneous and included newly diagnosed patients (94.9%) or patients relapsing after radical prostatectomy or radiotherapy (5.1%), who had either high-risk localized prostate cancer (26.6%), node-positive non-metastatic disease (N1M0, 19.2%), or metastatic disease (M1, 49.1%). OS was significantly increased with the addition of abiraterone (three-year OS: 83% versus 76%, HR 0.63, 95% CI 0.52–0.76). The benefit was seen in both, non-metastatic and metastatic disease (HR 0.75 and 0.61, respectively) [33].

### *b3. ADT plus new generation anti-androgens*

Three randomized trials (TITAN, ARCHES and ENZAMET) compared ADT plus new generation anti-androgens over ADT alone for mHNPC. In the TITAN trial, 1052 patients were randomly assigned to ADT plus either apalutamide (240 mg daily) or placebo. Approximately 11% had received prior docetaxel, but concurrent use of docetaxel was not permitted. At a median follow-up of 23 months, OS was longer with apalutamide (two-year OS 82% versus 74%, HR 0.67, 95% CI 0.51–0.89). Benefit was seen in men with high-volume (63%) and with low-volume metastatic disease (37%) [34]. ARCHES randomized 1150 mHNPC patients to ADT plus enzalutamide 160 mg daily or placebo. Patients were stratified by disease volume and prior docetaxel therapy. Enzalutamide significantly improved rPFS (HR 0.39; 95% CI 0.30–0.50; *p* < 0.001). Final OS results have not been published yet [35]. The ENZAMET study randomized 1125 men with mHNPC to either ADT plus other non-steroidal anti-androgens, (bicalutamide, nilutamide or flutamide), versus ADT plus enzalutamide. Enzalutamide showed a significant improvement in OS (HR 0.67; 95% CI 0.52–0.86); 45% of patients were planned to receive docetaxel. There was no OS benefit when the analysis was restricted to patients who received docetaxel and concurrent enzalutamide (three-year OS 73% versus 74%) that was even associated with more docetaxel toxicity [36].

According to the results of several meta-analyses, no clear advantage of one regimen over the others can be found in terms of OS [37–40]. The choice of the specific regimen should be discussed with the patient taking into consideration the potential toxicities associated, duration of treatment, comorbidities, preferences, and cost.

### *b4. Radiotherapy in mHNPC*

The impact of concurrent local prostate radiotherapy with ADT has been also tested in two randomized trials. The phase III HORRAD trial assigned men with primary mHNPC, bone metastases PSA > 20 ng/mL to ADT with or without external beam radiotherapy. Two-thirds of the men had more than five bone metastases. At a median follow-up of 47 months, median OS was not improved by the addition of radiotherapy, although some benefit could be found in the subgroup of patients with fewer than five metastases [41]. The STAMPEDE trial allowed docetaxel in both arms in addition to ADT, and radiotherapy to the primary started within 3–4 weeks after docetaxel. In the whole population, radiotherapy improved failure-free survival (HR 0.76; 95% CI 0.68–0.84; *p* < 0.0001) but not OS (HR 0.92; 95% CI 0.80–1.06). The prespecified low-volume subgroup (CHAARTED criteria), had a significant benefit in both failure-free survival (HR 0.59; 95% CI 0.49–0.72) and OS (HR 0.68; 95% CI 0.52–0.90) [42].

### Recommendations:

- In high-risk or high-volume mHNPC patients, ADT should be combined with docetaxel, abiraterone, enzalutamide or apalutamide, rather than using ADT alone (I, A).
- In low-volume mHNPC patients, RT to the primary tumor combined with systemic therapy is recommended (I, B).

## Definition of castration-resistant prostate cancer (CPRC).

- Definition of castration-resistant prostate cancer.
  The Prostate Cancer Clinical Trials Working Group 2 (PCWG2) defines CRPC as the state of castrate serum levels of testosterone (< 50 ng/dl or 1.7 nmol/l) plus biochemical or radiological progression as defined by the PCWG2 [43], despite anti-androgen withdrawal for at least 4–6 weeks.
  The CRPC state can be categorized as either metastatic (mCRPC) or nonmetastatic (nmCRPC). NmCRPC ultimately evolves to mCRPC and the average duration of this transition from ADT initiation is 19 months [44].
- Definition of non-metastatic castrate-resistant prostate cancer (nmCRPC).

NmCRPC is defined as PSA progression occurring despite treatment with primary ADT and in the absence of obvious disease in conventional imaging [45]. The PCWG-3 definition of nmCRPC includes: a minimum PSA level of 1.0 ng/ml, a rising PSA that is at least 2 ng/ml higher than the nadir PSA, castrate levels of testosterone, and no radiographic or bone scan evidence of metastases [46]. Without treatment, median bone metastasis-free survival (MFS) in nmCRPC ranges between 25 and 30 months, and 70% of patients remaining bone-metastases-free after 2 years. Baseline PSA level, PSA velocity and PSA-DT have been associated with bone metastasis-free and OS. These factors may be used to guide follow-up. The RADAR consensus statement (Radiographic Assessments for Detection of Advanced Recurrence) suggested a bone scan and a CT scan when the PSA reached 2 ng/ml. If negative, it should be repeated with PSA ≥ 5 ng/ml, and then after every doubling of PSA based on PSA testing every three months [47]. MRI has a low sensitivity when PSA is below 2 ng/ml, however, PSMA PET/CT has been shown to identify cancer in 33%, 46%, 57%, 82%, and 97%, in men with post-RP PSA ranges of 0–0.19, 0.2–0.49, 0.5–0.99, 1–1.99, and > 2 ng/ml, respectively. New imaging techniques will likely enable earlier and more accurate diagnosis of subclinical metastatic disease [48].

### Recommendations:

- Standard of care imaging for patients with nmCRPC is still conventional CT and bone scan. Perform new imaging techniques if results may condition the therapy (III,C).
- Risk stratification can define the frequency of imaging (III;B)**.**

## Therapeutic options for nmCRPC

1. ADT and first-generation anti-androgens
  Continuing ADT is the most recommended strategy for nmCRPC patients [49]. A 2 to 6-month median OS advantage was reported in nmCRPC patients who were castrated compared to those discontinuing ADT once CRPC developed. In addition, all subsequent treatments have been developed in men with ongoing ADT. Although first generation anti-androgens have been used in the past, no trial showed any benefit in terms of survival or quality of life, and they are not exempt of toxicity [22]. In addition, rotating LHRH agonist does not provide any clinical benefit.
  Recommendation:
  ADT should be continued in patients with CRPC (III, C).
2. New generation AR targeted therapy

There were no EMA-approved treatments for nmCRPC until the recent results of several pivotal double-blind multicenter phase III randomized trials that compared ADT plus placebo versus ADT plus enzalutamide (PROSPER) [50], apalutamide (SPARTAN) [51], or darolutamide (ARAMIS) [52]. In all trials, patients were of high risk (PSADT ≤ 10 months), and detection of metastases was done by conventional imaging with computed tomography (CT) scans of the chest, abdomen, pelvis, as well as technetium bone scans. In these trials, metastases-free survival (MFS) was the primary endpoint and OS was a secondary objective.

The SPARTAN trial included 1207 nmCRPC patients. MFS was significantly better in apalutamide-treated patients (40.5 vs. 16.2 m, HR: 0.28, 95% CI 0.23–0.35; *p* < 0.001) [51]. Symptomatic progression was also significantly improved in those who received apalutamide (HR of 0.45, 95% CI 0.32–0.63, and *p* < 0.001). However, several adverse events (AEs) of interest are noteworthy, including a higher incidence of some adverse effects in the apalutamide arm compared to the placebo arm, such as rash (23.8% versus 5.5%), hypothyroidism (8.1% versus 2%), and fractures (11.7% versus) 6.5%. At the final OS analysis with a median follow-up of 52 months, a significant increase in median OS with apalutamide + ADT vs. placebo + ADT was reported (73.9 vs. 59.9 months, HR: 0.784) [53].

The PROSPER trial randomized 1401 nmCRPC patients (2:1). Enzalutamide significantly prolonged median MFS (36.6 m vs 14.7 m [*p* < 0.0001]), time to first use of new antineoplastic therapy (39.6 m vs 17.7 m [*p* < 0.0001]), and time to PSA progression (37.2 m vs 3.9 m [*p* < 0.0001]) compared with placebo [50]. In the final OS analysis, median OS was 67.0 months in the enzalutamide arm and 56.3 months in the placebo arm (HR 0.73; 95% CI 0.61–0.89; *p* = 0.0011). Adverse events were higher with enzalutamide vs placebo (any grade adverse effects: 87% vs 77%; grade ≥ 3 adverse effects: 31% vs 23%; serious adverse effects: 24% vs 18%) [54]**.**

The ARAMIS trial included 1502 nmCRPC patients. Median MFS was 40.4 months with darolutamide vs 18.4 m with placebo (HR 0.41, 95% CI 0.34–0.50) [52]. At the final analysis of OS, darolutamide also showed a statistically significant OS benefit (HR 0.69, 95% CI 0.53–0.88, *p* 0.003), as did time to pain progression, (40.3 vs 25.4 m, HR 0.65, 95% CI 0.53–0.79; *p* < 0.0001). The rate of grade 3–5 adverse events was similar between the two groups with 24.7% in the darolutamide arm vs. 19.5% in the placebo arm [55].

Recommendation:

- For patients with high-risk nmCRPC ADT plus enzalutamide, darolutamide or apalutamide prolong MFS and OS. Selection of systemic therapy should be based on toxicity profile and an overall strategy. In view of the long-term treatment with these AR targeted agents in asymptomatic patients, potential adverse events need to be taken into consideration and the patient informed accordingly (I, A).

## Criteria for selecting the therapeutic sequence in mCPRC

First-line treatment for mCRPC should be decided considering previous treatments and the population of patients that were included in the available trials.

According to the TAX327 study [56], docetaxel improves symptoms and OS of patients who progressed to ADT. Symptomatic patients should receive docetaxel as first line, except in case of contraindication (i.e., hypersensitivity or high risk for toxicity). An increase in OS and symptoms relief has also been demonstrated by adding abiraterone–prednisone or enzalutamide to ADT in asymptomatic or mildly symptomatic patients [57, 58]. No direct comparison between docetaxel, abiraterone, and enzalutamide has been performed to date. Patients with visceral metastases were only included in the pivotal trials of docetaxel and enzalutamide.

Abiraterone–prednisone [59], enzalutamide [60] and cabazitaxel (CBZ) [61] have shown OS benefit in randomized trials after docetaxel. For mCRPC patients who have previously received any new generation AR targeted therapy, the sequence of a different hormone drug failed in demonstrating a survival benefit [62, 63]. Based on the results of TAX327, it seems reasonable to indicate docetaxel in this scenario.

The strongest evidence for a third line after docetaxel and a new generation AR targeted therapy comes from the CARD trial that compared cabazitaxel versus the new generation anti-androgen not previously administered [64]. Significant benefit was demonstrated both in PFS (HR: 0.54; 95% CI 0.40–0.73; *p* < 0.001) and OS (HR 0.64; 95% CI 0.46–0.89; *p* = 0.008]. According to these results, in absence of contraindication, cabazitaxel should be the third line of choice. In case of contraindication for chemotherapy, exclusive bone metastases and symptomatic disease, radium-223 may be considered, since OS benefit has been demonstrated in mCRPC [65]. The role of new targeted therapies in the sequence is discussed below.

Recommendations:

- Docetaxel—prednisone should be the first option for symptomatic patients who have received ADT alone. For asymptomatic or mildly symptomatic patients, docetaxel, abiraterone–prednisone or enzalutamide are recommended (I, A)
- In mCRPC patients who have progressed to docetaxel, abiraterone–prednisone, enzalutamide or cabazitaxel are recommended (I, A).
- In mCRPC patients who have progressed to a new generation anti-androgen therapy docetaxel-prednisone are recommended (I, B).
- Cabazitaxel is indicated as third line after a sequence of docetaxel and an androgen-signaling-targeted inhibitor (I, A).
- In mCRPC patients with symptomatic bone metastases and contraindication or progression to docetaxel, radium-223 may be considered (I, B).

## Aggressive variants

Adenocarcinoma is the most frequent histology in prostate cancer with other histologies including small-cell neuroendocrine carcinoma accounting for 1%.

Aggressive variants of prostate cancer are increasingly recognized in the clinic, probably as consequence of a greater survival and the pressure of the new hormonal treatments on the androgen receptor (AR).

Neuroendocrine prostate cancer (NEPC) comprises a wide range of situations among pure small-cell carcinomas and aggressive variant prostate cancers (AVPC), that can arise de novo (< 1% of cases), or much more commonly as intermediate variants after hormonal therapy for prostate adenocarcinoma. In a multi-institutional prospective study of 202 consecutive patients, 73% with prior progression to abiraterone and/or enzalutamide, the incidence of NEPC was 17% [66].

Small-cell NEPC is an aggressive subtype characterized by small, round, blue neuroendocrine cells, which do not express AR or secrete PSA, but usually express neuroendocrine markers (chromogranin A, synaptophysin and NSE) [67]. NEPC frequently metastasizes to visceral organs, and responds only transiently to platinum-based chemotherapy, with a median OS of less than one year. The AVPC share clinical, biological, and response profiles with androgen-indifferent, platinum-sensitive small-cell carcinomas but maintaining histological features of undifferentiated carcinoma, and harboring defects in tumor suppressors genes such as *TP53*, *RB1*, and *PTEN* [68]. According to the most accepted definition of AVPC must have at least one of the clinical characteristics showed in Table 2.

**Table 2:** AVPC clinicopathological characteristics

1. Histological evidence of small-cell prostate carcinoma
2. Exclusively visceral metastasis
3. Predominantly lytic bone metastasis
4. Bulky (> 5 cm) lymphadenopathy or primary tumor with Gleason score of 8 or more at diagnosis
5. Low PSA (< 10 ng/ml) plus high volume (≥ 20) bone metastasis at initial presentation
6. Elevated (> 2 × ULN) LDH or CEA
7. Short interval response (< 6 months) to androgen deprivation therapy

AVPC is sensitive to platinum-based chemotherapies. In a single-arm sequential phase 2 trial, 120 patients with mCRPC and at least one of the seven prior criteria (including neuroendocrine markers on histology or serum) were treated with first-line carboplatin—docetaxel (CD) followed by second-line etoposide—cisplatin (EP). PFS after four courses of CD and EP were 65.4% and 33.8%, respectively. The median OS was 16 months (95% CI 13.6–19.0 m). Neuroendocrine markers did not predict outcome or response to therapy [69].

The same group recently published a phase 1–2 randomized trial of cabazitaxel vs the combination of carboplatin and cabazitaxel in 169 patients with progressive mCRPC, 56% of which met at least one of the prior criteria for AVPC [70]. A maximum tolerated dose of cabazitaxel at 25 mg/m^2^ and carboplatin of AUC 4 was selected for phase 2. Pre-specified subgroup analysis of PFS showed that the combination favored only patients with AVPC criteria (HR 0.58, 95% CI 0.37–0.89, *p* = 0.013).

Recommendation:

- Platinum-based chemotherapy should be considered the first option in mCRPC with clinicopathological characteristics of AVPC (II, B).

## New strategies in metastatic prostate cancer (MPC)

Given the prevalence of inactivating mutations in genes involved in DDR pathways such as the Homologous Recombination Repair (HRR) pathway [71], MPC may be sensitive to ADP-ribose polymerase (PARP) inhibition by a mechanism of “synthetic lethality” [72]. The randomized phase III double blind PROfound trial compared the PARP inhibitor olaparib versus abiraterone or enzalutamide in mCRPC patients with deleterious alterations in at least one of 15 genes involved in DDR, and previous treatment with one of these two AR signaling inhibitor. In this study, 28% of the 2792 samples analyzed harbored an alteration in the HHR pathway. *BRCA2* was the gene most frequently altered (8.7%) followed by *CDK12* (6.3%), *ATM* (5.9%), *CHEK2* (1.2%), and *BRCA1* (1%). In cohort A (patients with *BRCA1, BRCA2* or *ATM* alterations), a significant benefit was observed for olaparib in rPFS (7.4 vs 3.6 months; HR 0.34; 95% CI 0.25–0.47). A longer OS was also observed (median OS 18.5 vs 15.1 months; HR: 0.64, 95% CI 0.43–0.97), despite crossover to olaparib in 66% of patients [73, 74] In the single-arm, phase II TRITON2 trial, Rucaparib showed a 43.5% ORR and 54.8% PSA response rate in *BRCA1/BRCA2* mutated mCRPC patients progressing after AR inhibitors and docetaxel [75]. Both olaparib and rucaparib have received FDA approval, and olaparib EMA approval for *BRCA1/2*-mutated patients in the mCPRC setting, so that *BRCA1* or *BRCA2* mutations, might be the first predictive biomarker implemented in the clinic. While benefit in *BRCA2* mutated patients is well-established, the role of mutations in other genes such as *ATM, PALB2* or *CDK12* warrant further study. Evidence from retrospective studies also suggest that platinum therapy could confer clinical benefit in DDR deficient prostate cancer [76].

Loss of *PTEN* results in activation of the AKT pathway. In the phase III IPATential 150 trial, the combination of abiraterone and the AKT inhibitor ipatasertib showed a benefit in rPFS compared to abiraterone (HR 0.77, 95% CI 0.61–0.98; *p* = 0.033) in patients with PTEN loss as defined by IHC. Further follow-up and data on the impact on OS will be necessary to elucidate the role of Akt inhibitors in mCRPC.

Although prostate cancer was one of the first diseases where an immunotherapeutic agent was approved (sipuleucel-T) [77], this strategy remains investigational. Pembrolizumab achieves ORR of 5–10% [78]. This drug has been approved for tumors MMR deficiency or MSI independent of the tumor type, accounting for 3–8% of MPC.

Theragnostics, defined as the combination of a predictive biomarker with a therapeutic agent [79] is another still investigational promising strategy. Some trials with radioligands and PSMA a type II transmembrane overexpressed on prostatic cancer cells [80] are ongoing to clarify the role of RLT in the treatment algorithm of mCRPC [81].

Recommendations:

- Olaparib is recommended in *BRCA1/BRCA2* mutated mCRPC patients after progression on at least one new generation AR targeted therapy [I, A]
- Immune checkpoint inhibitors may be considered in patients with microsatellite instability or mismatch repair deficiency [II, B]
- Currently, insufficient evidence is available in mCRPC to recommend Akt inhibitors [I, C] or radioligand therapy [II, B].

## Criteria for genetic testing

Importantly, half of the DDR mutations identified in prostate tumors are also present in the germline [13] and may represent an inherited cancer predisposition. Early identification of mutation carriers is relevant for cancer screening and early detection programs. Genetic testing has traditionally been based on an early age at diagnosis and a strong family history of cancer, however, such family background is absent in a third of prostate cancer patients found to carry a germline mutation [82–84]. Therefore, experts and major cancer organizations recommend germline testing for all patients with metastatic prostate cancer, regardless of tumor characteristics or family history [85–87]. The second Philadelphia Prostate Cancer Consensus Conference recommends the use of a comprehensive gene panel that should include *BRCA1, BRCA2* and MMR genes (*MSH2, MLH1, PMS2, MSH6* and *EPCAM)*. The inclusion of ATM and other genes linked to cancer predisposition syndromes should be considered on the bases of personal or family history of cancer. Germline testing is also recommended for patients with pathogenic/likely pathogenic BRCA2 mutations identified through tumor sequencing and should also be considered in patients with alterations in other genes linked to cancer predisposition syndromes (i.e., *BRCA1, ATM*, MMR genes). Genetic counseling should always be offered before ordering the test [88]. During this process, healthcare providers should discuss with patients the purpose of genetic testing; potential types of test results; the possibility of uncovering hereditary cancer syndromes and additional cancer risks as well as addition familial testing. Clinicians without specific training or expertise should refer patients to genetic counseling before ordering germline testing.

Recommendations:

- Germline testing for *BRCA1/2* and other genes associated with cancer predisposition syndromes is recommended for patients with a family history of cancer [I,A] as well as in all patients with metastatic prostate cancer [III, A].
- Patients with somatic pathogenic/likely pathogenic mutations in genes linked to cancer predisposition syndromes (*i.e., BRCA2)* should undergo germline testing [III, A].
- Genetic counseling by clinicians with specific training or expertise should always be offered before ordering germinal testing [III, A]
